# A study of sex difference in infant mortality in UK pediatric intensive care admissions over an 11-year period

**DOI:** 10.1038/s41598-021-01173-x

**Published:** 2021-11-08

**Authors:** Ofran Almossawi, Scott O’Brien, Roger Parslow, Simon Nadel, Luigi Palla

**Affiliations:** 1 Department of Population, Policy and Practice, GOS UCL Institute of Child Health, London, WC1N EH1 UK; 2 Pharmacy Department, Great Ormond Street Hospital, London, UK; 3 Paediatric Intensive Care Unit, Imperial College Healthcare NHS Trust, London, UK; 4 Leeds Institute of Cardiovascular and Metabolic Medicine, University of Leeds, Leeds, UK; 5 Department of Public Health and Infectious Diseases, University of Rome La Sapienza, Rome, Italy; 6 Department of Medical Statistics, London School of Hygiene & Tropical Medicine, London, UK; 7 School of Tropical Medicine and Global Health, Nagasaki University, Nagasaki, Japan

**Keywords:** Paediatrics, Paediatric research, Paediatric research, Medical research, Epidemiology

## Abstract

Within the UK, child mortality from all causes has declined for all ages over the last three decades. However, distinct inequality remains, as child mortality rates are generally found to be higher in males. A significant proportion of childhood deaths in the UK occur in Paediatric Intensive Care Units (PICU). We studied the association of sex with infant mortality in PICUs. We included all infants (0 to 12 months old) admitted to UK PICUs from 01/01/2005 to 31/12/2015 using the Paediatric Intensive Care Audit Network (PICANet) dataset. We considered first admissions to PICU and fitted a cause-specific-hazard-ratio (CSHR) model, and a logistic model to estimate the adjusted association between sex and mortality in PICU. Pre-defined subgroups were children less than 56-days old, and those with a primary diagnosis of infection. Of 71,243 cases, 1,411/29,520 (4.8%) of females, and 1,809/41,723 (4.3%) of males died. The adjusted male/female CSHR was 0.87 (95%-CI 0.81 to 0.92) representing a 13% higher risk of death for females. The adjusted OR for male to female mortality is 0.86 (95%-CI 0.80 to 0.93). Analyses in subgroups yielded similar findings. In our analysis, female infants have a higher rate of PICU mortality compared to male infants.

## Introduction

Child and infant mortality are important indicators of child health. The September 2010 United Nations Summit set a target for the fourth millennium development goal (MDG) to reduce the mortality rate of children under five years of age by two thirds between 1990 and 2015^1^. Over the past three decades, there have been sizeable reductions in infant and child mortality throughout resource rich countries mainly because of improvements in obstetric, pediatric medical and surgical care, and positive public health initiatives. Within the UK alone, child mortality from all causes has declined over all ages by 50–70% between 1980 and 2010^2,3^. Despite this progress, distinct inequality remains, as child mortality rates vary by sex and are generally found to be higher in males^4^. The 2018 mortality rate per 100,000 for England and Wales show male–female mortality ratios of 1.21 at age under 1 year, 1.26 at age 1–4 years, 1.00 at age 5–9 years, and 1.57 at age 10–14 years^5^. A similar survival advantage for females is also observed in extremely premature neonates^6^ and historically has been hypothesized to be linked to a sex ratio at birth more in favour of males^7,8^.

Causes of child and infant mortality are complex and include biological, environmental, and behavioural influences. In the child and adolescent age group, the sex mortality difference is thought to be mainly related to external factors such as environmental and behavioural influences, while in previous studies neonates sex mortality differences have been ascribed to infection/sepsis and mortality has been found to be higher in males^9^. Our study though specifically focuses on UK infants (0-12 months) to expand the dataset and the analytical methods utilised in two previous analyses^14,15^ In the UK setting infant mortality is relatively low, with the death rate at 3.6 per 1,000 live births. With regard to infection in the UK, previous results indicate that mortality by infectious diseases in UK children between 0–4 years of age amounted to 63.9 per 100,000^10^. Over 65% of childhood deaths in the UK occur in hospitals^11^, and over 85% of these deaths (hence over 50% of all childhood deaths) occur in Paediatric Intensive Care Units (PICUs)^12^. With respect to the specific effects of infections in childhood, it has been suggested that the two sexes may have variable immune responses to infections as mediated by their sex-hormones^13^. Preliminary analyses of admissions of infants to UK PICUs due to infections reported larger numbers of male admissions over a two-year period, but a greater female than male infant mortality^14^ A follow up to this analysis was carried out over a five-year period and produced similar results^15^.

### Aims

We designed the present study with the primary aim to further investigate the difference in mortality within PICU between male and female infants admitted to PICUs in the UK. We limited the analysis to first admissions to PICU for reasons discussed further under methods. A secondary aim was to clarify the assumptions and data required to minimise the confounding bias that would affect the estimation of the sex-mortality association in PICU infants using observational data^14,15^.

## Methods

### Data source

We obtained anonymised data for all infant admissions to UK PICUs from the Paediatric Intensive Care Audit Network (PICANet) from 1st January 2005 to 31st December 2015. PICANet is a national audit database recording the demographic and clinical data of children admitted to UK PICUs. The Collection of personally identifiable data by PICANet has ethical approval and has been approved by the Health Research Authority Confidentiality Advisory Group. The data is collected by individual PICUs as daily activity data, or Paediatric Critical Care Minimum Data Set (PCCMDS)^16^. The data contains anonymised information on individual patients from all 35 UK PICUs. Also linked to this dataset was the Index of Multiple Deprivation (IMD) score^17,18^, a UK government qualitative measure of deprived areas.

Infants are admitted to a PICU and not a neonatal ICU if they had been discharged home after birth and then subsequently required critical care admission. Their initial discharge home could be from maternity services, or after a period of admission to a neonatal ICU.

### Description of variables

The relevant variables in this dataset were: age (in days, converted to 30 day intervals); primary diagnosis of infection; deprivation score (IMD) 2010); PIM2R score (Paediatric Index of Mortality-2 Recalibrated, 2016 recalibration) which is a paediatric risk of mortality score calculated on admission to PICU^19^, whether the patient received invasive ventilation and duration of invasive ventilation (in days); inotropic support; renal support; planned versus unplanned admission; gestational age (in weeks); and ethnicity (four categories). From primary diagnosis of infection, we selected out primary diagnosis of bronchiolitis, as this diagnosis is known to have a significantly lower mortality risk than other infections^20,21^. We did not have information on comorbidities or chronic conditions. However the PIM2R score captures some of the variability relating to chronic conditions as it incorporates information on high and low risk diagnoses.

### Statistical methods

#### Descriptive statistics

For all analyses, we define the study population as the first admission to PICU only. For infants with multiple admissions during the time period of the study, only the first admission is included.

For descriptive analysis, we used proportions and ratios to summarise categorical data, and means, standard deviations for numerical data or medians and first/third quartiles for very skewed distributions of numerical variables. As a descriptive summary of mortality, we fitted an unadjusted and PIM2R-adjusted logistic model for each PICU to describe the variability of the outcome between PICUs. To estimate the overall PIM2R-adjusted odds ratio, we fitted a logistic model with cluster robust standard errors (to account for clustering by PICU). We removed children who were still in PICU after 100 days (n = 192) as these were considered to have an atypical Length of Stay (LOS). The median LOS for a PICU stay in infants up to one-year of age (in the period 2014 to 2016) was 4.8 days (PICANet 2017 report). As the dataset for our study spans 11 years over which medical techniques in pediatric critical care have advanced, we generated a time-series plot to describe the mortality trends.

Observations where the sex or outcome were missing were removed. We excluded patients with a PICU LOS of less than two hours who were discharged alive, as these were not considered to be true PICU stays^2^. For children with multiple PICU admissions, only the first admission was retained in the dataset to reduce any bias resulting from correlated data (since multiple admissions are indicated but not linked in the dataset). And finally, observations with missing LOS data were removed due to lack of time to event information.

To determine that our mortality ratios by sex are consistent with the published mortality rate ratio in the general population of children of that age, we used Bayes theorem to work out the posterior sex probability ratio (odds) for those who die in PICU by deriving the respective conditional posterior probabilities.

#### Variable relationships using DAGs

We drew directed acyclic graphs (DAGs) that represented the hypothesised causal relationships between the variables (see Supplemental Digital Content^22^) and showed under which scenarios we expect to derive valid estimates (these are detailed in the Supplemental Digital Content).

The aim of the DAGs are to clarify the relationship between sex and mortality in PICU, in the presence of other causally linked variables. This is to ensure the correct adjustments are made in the analysis to avoid inducing a false association between sex and death in PICU. With the current dataset, it is not possible to make any causal statements, rather we expect through the use of the DAGs to avoid bias induced through adjustment.

**Figure 1:**
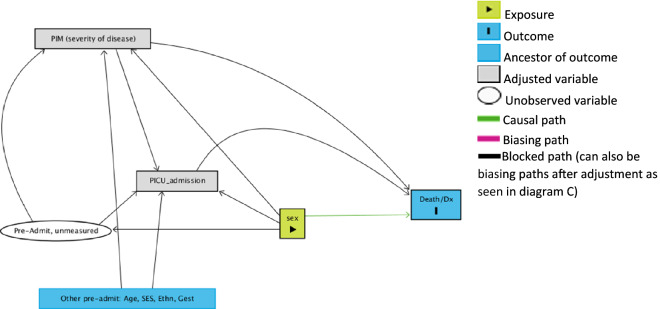
Relationship between variables after the additional adjustment for PIM2R. This figure shows that adjusting for PIM and not adjusting for age, ethnicity, SES, and gestation would not bias the relationship between sex and mortality in PICU, given the stated assumptions. Please refer to the Supplementary Digital Content for an expanded study of the relationships between variables.

In summary, the DAG assumptions we made in Fig. 1. (also reproduced as DAG C in Supplemental Digital Content) indicated that, in order not to bias the relationship between sex and mortality in PICU, we had to adjust our statistical models for the variable PIM2R.

The information incorporated in the graph may be incomplete and therefore the DAGs should be interpreted with caution. In particular our DAGs are based on a number of assumptions:

1. There is a different relationship between sex and factors potentially affecting admission to PICU in that here measured factors (age at admission, socioeconomic status—here measured by the IMD score, ethnicity) are not directly caused by sex (no directed arrow from sex into them) while some of the unmeasured factors (maternal and perinatal factors such as maternal illness during pregnancy, or complications during child birth or admission/contact with other health facilities) are caused by sex (directed arrow from sex).
2. There is no direct arrow between either measured and unmeasured preadmission factors and death in PICU, as these factors are assumed to be directly linked to admission to PICU and be related to death only by influencing admission. (For the alternative scenario where the direct arrow between measured and unmeasured preadmission factors and death in PICU is present, please refer to the Supplemental Digital Content).
3. We have not included any time-varying (within PICU) variables such as information on duration of inotropic and renal support as we did not have information on their duration. Furthermore, adjustment for the post admission variables may bias the results in that these variables are downstream from the admission and may be on the causal pathway. In such cases, other analyses such as mediation analysis may be needed which is beyond the scope of this paper.

#### Time-to-event analysis

**Figure 2:**
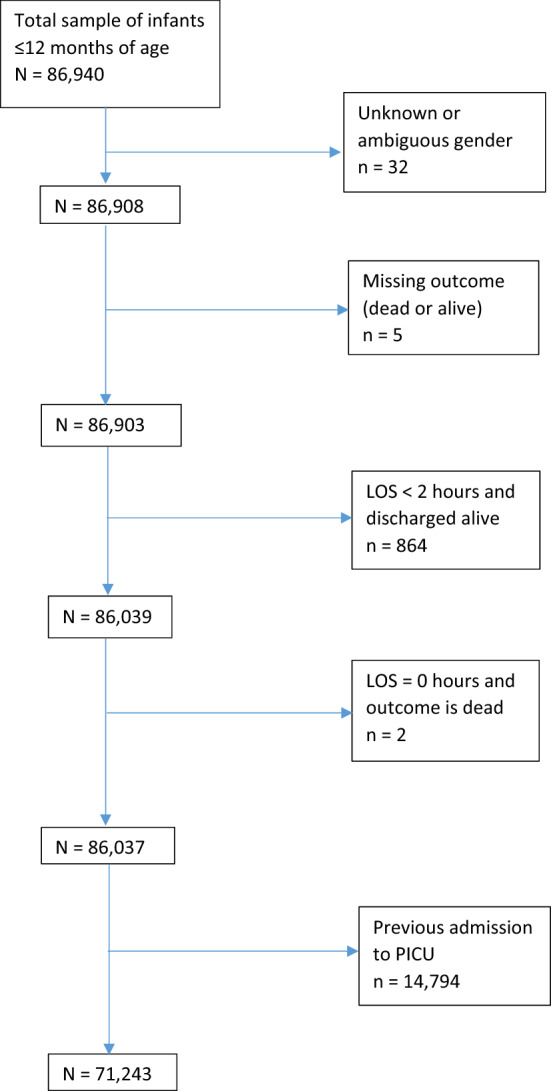
Data preparation.

Death within PICU and discharge from PICU (either to home or to another hospital) are two mutually exclusive competing risks. As the dataset had very little censoring (only five missing outcomes, see Fig. 2) we used a logistic regression model for PICU mortality at 100 days as the main analysis, and additionally used a cause specific hazard model (CSHR) censored at 100 days to investigate possible time trends within those 100 days.

We assessed the assumption of proportionality of hazard (time varying coefficients), after fitting a Cox proportional hazard model including the selected adjustment variables, by plotting the Schoenfeld residuals for all the variables in the model. We carried out further assessments of proportionality using parametric restricted cubic splines, to better characterise the variability of the hazard ratios over time for all the variables in the model, via the Stata post estimation command STPHCOXRCS^23^. We used clinical justifications to decide on the placement of the internal knots at 2, 5, and 10 days. We fitted the final CSHR model using the variables selected though the DAG analysis (sex and PIM2R). We included an interaction of splines with time for the variables sex and PIM2R, each with three internal knots placed at (2, 5, and 10 days). We assessed the functional forms of continuous variable (PIM2R) using Martingale residuals. All steps above were carried out using cluster robust standard errors to account for unobserved variability between PICUs.

#### Logistic regression

For logistic regression analysis we assessed the functional forms of the continuous variables using lowess plots. We then fitted a logistic model including death as outcome, sex as expsoure and PIM2R as an adjustment variable, based on the study of variable relationships (Figure 1 DAG). A further logistic regression model was fitted with the complete cases only and adjusted for PIM2R, IMD score, ethnicity, and gestational age. Length of stay in PICU is incorporated in the model by using time-to-event analysis, but otherwise we did not include this as an adjustment variable in the logistic model.

#### Subgroup analyses

Our predefined subgroup analyses were to test the hypotheses that sex mortality differs within the subgroups of children with a primary diagnosis of infection, and in children under 56 days (at PICU admission), which was presumed to be before their first routine infant vaccination.

To identify patients with a primary diagnosis of infection, ICD10 codes were used. Infants admitted with a diagnosis of bronchiolitis were not included in the infection category.

### Ethics approval

London School of Hygiene & Tropical Medicine, MSc Ethics Ref: 10,684.

### Consent

The data extract used was anonymised and no additional consent was required. The Collection of personally identifiable data for the PICANet project has been approved by the Patient Information Advisory Group (now the Health Research Authority Confidentiality Advisory Group) and ethical approval granted by the Trent Medical Research Ethics Committee, ref. 18/EM/0267.

## Results

During the time period of the study, there were 86,037 PICU admissions. After keeping only the first admission for each infant, 14,794 admissions (17.2%) were removed. Therefore, after data preparation, 71,243 infants were included in the final analysis. Figure 2 summarises the characteristics of the excluded individuals. Of the 71,243 infants 43,488 (61%) had LOS of four days or less and out of 3,220 deaths, 1,677 (52%) occurred in the first four days.

**Figure 3:**
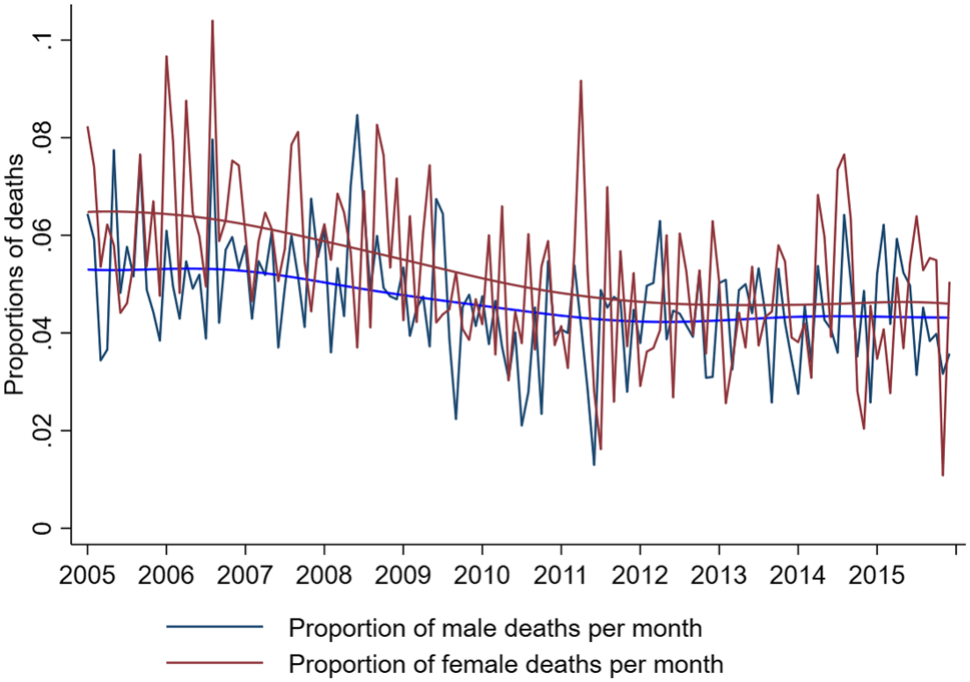
Time series plots of death for males and females. The jagged lines are the variations in total deaths for males and females by month. The lowess smooth lines are the overall totals for average deaths per month for males and females over the 11-year study period, showing overall decline (smooth curves) in mortality.

The overall summary of baseline characteristics is presented in Table 1. Out of all PICU admissions 58.6% were males. The distributions of clinical characteristics were very similar for the two sexes. There were 1,411/29,520 (4.8%) female deaths, and 1,809/41,723 (4.3%) male deaths. There were numerically more male deaths than female deaths because more males were admitted than females. However, as Fig. 3 shows, the proportion of females who died was consistently higher than males in every year, over the 11 year study period.

**Table 1:** Baseline demographics by sex.

	Female (n = 29,520) (41·4%)				Male (n = 41,723) (58·6%)				Total (N = 71,243)			
	n*	Mean or %	Median	IQR	n*	Mean or %	Median	IQR	n*	Mean or %	Median	IQR
Age /days	29,520	102.51	67.00	14–169	41,723	98.84	60.00	13–161	71,243	100.36	63.00	14–164
Length of stay /days	29,514	5.66	2.98	1.21–5.96	41,720	5.71	2.96	1.18–5.95	71,234	5.69	2.97	1.19–5.96
Deprivation score	25,105	27.83	24.54	13.20–40.10	35,622	27.45	24.24	12.86–39.52	60,727	27.61	24.37	13.02–39.75
PIM2R (2016)	29,520	0.05	0.02	0.01–0.05	41,723	0.05	0.02	0.01–0.05	71,243	0.05	0.02	0.01–0.05
Duration of ventilation /days	18,641	4.95	3.00		26,552	5.01	3.00		45,193	4.98	3.00	
Ventilated during admission (%)	18,641	79.28%			26,552	77.70%			45,193	78.35%		
Planned admission (%)	29,485	40.22%			41,662	38.01%			71,147	38.92%		
Primary diagnosis bronchiolitis (%)	29,432	12.44%			41,625	12.53%			71,057	12.49%		
Primary diagnosis other infection (%)	29,432	11.37%			41,625	11.73%			71,057	11.58%		
Inotropic support during admission (%)	29,519	24.20%			41,723	23.53%			71,242	23.81%		
Renal support during admission (%)	28,577	3.53%			40,254	3.76%			68,831	3.67%		
**Gestation**												
23–27 weeks	1,782	7.88%			2,447	7.59%			4,229	7.71%		
28–35 weeks	4,297	19.01%			6,438	19.96%			10,735	19.57%		
36–44	16,522	73.10%			23,367	72.45%			39,889	72.72%		
Total	22,601				32,252				54,853			
**Ethnicity**												
White	17,741	76.35%			25,143	77.10%			42,884	76.79%		
Black	1,297	5.58%			1,514	4.64%			2,811	5.03%		
Asian	2,654	11.42%			3,796	11.64%			6,450	11.55%		
Mixed/Other	1,543	6.64%			2,158	6.62%			3,701	6.63%		
Total	23,235				32,611				55,846			

*n is the number of cases with non-missing data. Where n is different to that in the main header, it indicates missing data; PIM2R (2016): Paediatric Index of Mortality version 2 revised and recalibrated in 2016; Ventilated: refers to invasive ventilation.

**Figure 4:**
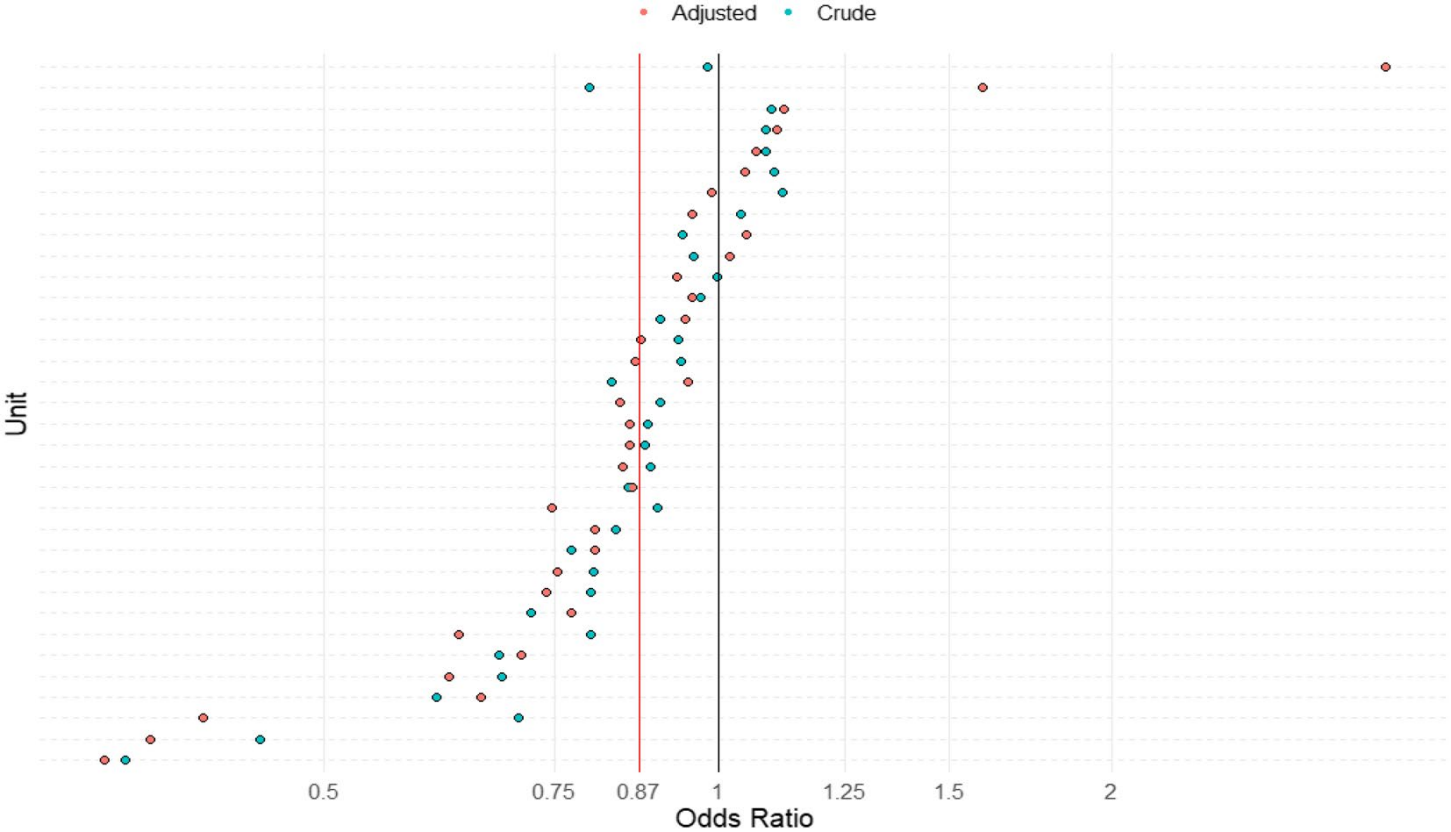
Crude and PIM2R adjusted mortality of males versus females for each critical care unit. The red vertical line is the overall PIM2R adjusted odds ratio from a cluster-robust model and the black vertical line is the null odds ratio value of one. The overall OR for male relative to female mortality is 0·87 (95% CI, 0·81 to 0·94, P = 0·001). Each dot represents an individual unit. OR < 1 represents higher female mortality in that PICU, OR > 1 represents higher male mortality. The PIM2R adjusted OR ensures that mortality comparison between the two sexes takes into account that the probability to be admitted in PICU depends on the severity of disease on admission. This plot can be viewed in conjunction with Table 4.

Both sexes showed a reduction in percentage mortality over the study decade, with females showing a sharper decline than males. The Crude and PIM2R adjusted odds ratios for mortality, per PICU and overall, are presented in Fig. 4.

The final variables we included in the time-to-event analysis and logistic regression were sex and PIM2R (converted to percentage risk of death). After running a Cox proportional hazard (CPH) model, the Schoenfeld residual plots and the flexible parametric post-hoc diagnostic plots showed a proportional hazard ratio for sex, and a time varying hazard ratio for PIM2R. The male to female CSHR was 0.87 (95% CI 0.81 to 0.92) representing a 13% higher risk of death for females over males.

The final CSHR model, using splines (to model the non-proportional effects of PIM2R), and cluster robust standard errors, is presented in Table 2.

**Table 2:** Parameters from the Cause-Specific Hazard Ratio model.

	CSHR	SE	P	95% CI	
Sex (male/female)	0·865	0·028	< 0·001	0·812	0·922
PIM2R	1·044	0·001	< 0·001	1·042	1·047
**Spline terms**					
Baseline 1	− 0·082	0·022	< 0·001	− 0·124	− 0·039
Baseline 2	− 0·135	0·020	< 0·001	− 0·175	− 0·095
Baseline 3	− 0·049	0·012	< 0·001	− 0·073	− 0·026
Baseline 4	0·132	0·016	< 0·001	0·100	0·165
PIM2R 1	− 0·008	0·001	< 0·001	− 0·010	− 0·007
PIM2R 2	0·003	0·001	< 0·001	0·002	0·004
PIM2R 3	− 0·001	0·000	0·034	− 0·002	0·000
PIM2R 4	− 0·002	0·001	< 0·001	− 0·003	− 0·001
Constant	− 5·038	0·076	< 0·001	− 5·187	− 4·888

*CSHR *Cause specific hazard ratio, *CI* Confidence interval, *P* p value, *SE* Standard error.

Table 3 shows the cumulative proportions of deaths and discharges at various intervals for the first 40 days of PICU admission. Female deaths are consistently higher than male deaths at each point.

**Table 3:** Marginal probabilities of death within PICU and discharge from PICU for males and females at different time points.

		Time in days				
		2	5	10	20	40
Female	Dead	0.018	0.028	0.034	0.040	0.045
Male	Dead	0.016	0.025	0.031	0.037	0.041
Female	Discharged	0.356	0.663	0.844	0.918	0.943
Male	Discharged	0.362	0.668	0.851	0.922	0.946

The parameters in Table 4 are from the logistic regression model with cluster robust standard errors to account for variations between the 35 PICUs. There is evidence for 14% lower odds of mortality for males compared to females.

**Table 4:** Parameters from the logistic regression model.

	OR	SE	P	95% CI	
Sex (male/female)	0.860	0.033	< 0.001	0.797	0.927
PIM2R	1.084	0.004	< 0.001	1.077	1.092
Constant	0.039	0.003	< 0.001	0.033	0.046

The OR of male to female deaths in Table 4 can also be compared to the time to event analysis (the CSHR). The complete case analysis of the logistic regression can be seen in Table S1 in the Supplementary Digital Content. The sample size was halved but the direction of the association remains in favour of higher female mortality. Coefficients for the other covariates in the complete case analysis are also presented in Table S1.

The subgroup analyses for logistic regression (Supplemental Digital Content) estimated a male to female OR of 0.84 (CI= 0.75 to 0.94) for children <56 days of age (Table S2) and of 0.88 (CI= 0.80 to 0.97) for children >56 days (interaction effect between age subgroups and sex had p=0.48 and CI = 0.81 to 1.10 (Table S4)). The subgroup OR was 0.87 (CI=0.73 to 1.04) for children with primary diagnosis of infection and 0.85 (CI=0.77 to 0.93) (Table S3) for children with primary diagnosis other than infection but including bronchiolitis (interaction effect with sex had p=0.92 and CI = 0.80 to 1.28) (Table S5). The results of time to event analysis (HR) were very similar (see Tables S6 to S9 in the Supplemental Digital Content, Adjustment variables for all the models are listed in each table).

We calculated the posterior sex probability ratio (odds) for those who die in PICU. See supplementary content for details of the calculations. Based on relevant probabilities within PICU (see the Supplemental Digital Content), we calculated the posterior odds of being female vs male among PICU deaths as 0.441/0.559 = 0.79 and hence in favour of females, consistently with the published mortality rate ratio in the general population of children of that age (= 0.82, according to National Statistics figures).

## Discussion

The increased numbers of male deaths over female deaths is well described in most analyses of mortality data in infants and children. However several recent studies have suggested greater female than male mortality in intensive care in children of all ages^24–27^. Given that a sizable proportion of childhood deaths in the UK occur in PICU, we deemed PICU admissions a relevant cohort in which to examine potential sex differences in infant mortality. Our study differs from previously published literature in that it is the largest study of infants in which the primary aim is to address the sex mortality difference in PICU, with careful consideration to the relationships between common variables and their relationships to the outcome. In particular, by drawing directed acyclic graphs connecting measured and unmeasured relevant variables, we clarified the data and conditions required to draw causal estimates of the direct effect of sex on mortality in PICU and highlighted the limitations of the current analysis.

The main finding from our analysis is that female sex is associated with higher mortality in PICU for infants up to 12 months of age for the first PICU admission and after adjustment for PIM2R score. This finding is supported by evidence from both of the analysis methods we have used. There was no evidence of effect modification by the subgroups of infants under/over the age of 56 days, thus suggesting no clear influence of primary immunisation, or of a primary diagnosis of infection on sex mortality differences. When compared to the relative mortality in the general population where male infants are reported to have higher mortality, both the CSHR and the OR are in the opposite direction. Higher odds/hazard of mortality for female infants in PICU persisted across all subgroups of infants.

Over the 11-year duration of the study we observed a decline in PICU mortality for both sexes, but with a sharper decline for females. However, the sex mortality difference between females and males that we have described remained.

Using simple probability calculations, the higher number of male admissions to PICUs, together with the lower rate of male infant deaths in PICU leads to posterior odds of 0.79 when assessing PICU deaths. This is still overall in favour of female survival and consistent with the mortality rate ratio calculated in the overall population of 0.82 based on published national statistics figures (ONS death summary tables of 2016, https://www.ons.gov.uk/) for under one year of age female to male mortality per 1000 live births. Hence the data we report do not contradict the numerically greater overall mortality of males (see calculations of posterior probabilities in Supplemental Digital Content). Rather, they warrant further investigation into the reasons for the greater proportion of males admitted to PICUs and the greater mortality of females in PICUs.

Our results are in line with four other studies, two of which indirectly investigated the effect of sex on mortality and others observing this effect as a secondary finding, in children with varying age ranges. These studies were carried out in the United States, 2011^24^, Spain, 2015^25^, Sweden, 2017^26^, and the UK^27^. The US study had a large sample size of over 80,000 cases which included children up to 18 years. It was a multi-centre study with over 31 PICUs and spanning 2005-2008. The focus of that study was to assess if ethnicity had an effect on PICU mortality and the effect of being female, which was only used as an adjustment confounder, was estimated as 12% higher odds of dying in PICU compared to males (p=0.019)^24^. The Swedish study analysed data for 21,972 children over an eight year period (2008-2015), and included all PICU admissions in Sweden of individuals <16 years old. They reported a sex difference in the HR of mortality of 0.91 for boys, p=0.035^26^. The Spanish single centre study of children 0–18 years admitted to PICU over a period of three years showed an in-hospital mortality advantage of males over females (3.3% versus 4.9%, p=0.042)^25^. The UK study, with a sample size of 154,667 and aged <18 years, investigated mortality in PICU for children with and without life-limiting conditions. Sex was used as an adjustment variable with a mortality odds ratio of 1.09 (p=0.002) for females compared to males^27^.

All these studies were conducted in high-resource settings with access to advanced critical care facilities for children. In these studies, gender was primarily used as an adjustment variable. Our current study confirms that the higher PICU mortality rate for females is consistent with that previously described over multiple healthcare settings i.e. socialised systems and private healthcare facilities. Strengths of our study are that it directly addresses the impact of sex on mortality in infants and it spans a longer duration than the other studies, with a primary focus on sex differences in mortality.

It is unclear what is driving the greater mortality risk for female infants in PICU. One hypothesis is that there are biological differences in the response to critical illness or in the response to interventions. Another implication from our study is that the quantification of severity of illness using PIM2R, which is calculated within the first hour of admission to PICU and is calculated independently of sex, is not calibrated for gender. If our analysis is replicated by other studies in the future, then it is probable that sex should be accounted for, either alongside the PIM score or within it. Since the PIM2R score had similar distributions between male and female infants, it is possible that female infants have a different trajectory of illness. Males and females may present with the same severity of illness, as suggested by the PIM score but at different stages of their illness, with males admitted to PICU earlier in the course of their illness.

## Limitations

Our study does not address any potential explanations for the observed sex difference in mortality as we did not have access to data factors that may influence PICU admission, such as maternal or birth factors. Moreover even for the available variables, the findings reflect the causal relationships we assumed between those variables as clearly stated in the DAGs.

PICANet data collection is carried out by individual PICUs and thus may vary. However, data collection is closely audited and sex and outcome are expected to be consistently recorded.

In addition, we did not have access to outcome after PICU discharge so it is not possible to assess if the mortality difference continues beyond discharge from PICU. Due the lack of linkage of repeated admissions of the same patients in the dataset, PICU admissions following the first one were not included in the analysis and therefore deaths in subsequent admissions would not be captured in the current study.

Our extract of PICANet data did not allow any analysis of potentially important co-morbidities that may influence PICU outcome. PIM2R captures information of certain underlying conditions, but this does not allow any specific analysis of these conditions and their influence on PICU outcome.

PICANet data also does not allow detailed analysis of specific organ failures. Therefore organ failure contributing to mortality cannot be analysed. However, our assumption is that mortality suggests unresolved organ failure, and discharge suggests that organ failure which required admission to PICU has resolved.

## Conclusion

We have shown evidence of greater relative mortality for female than male infants in PICUs across the UK. This persists after subgrouping by infection diagnosis and age prior to routine infant vaccination. This suggests that females and males may differ in the pathophysiology of critical illness and/or their response to treatment. The reasons for the disparity in outcome between females and males remains unclear and warrants further investigation in this and other populations.

## Supplementary Information

Supplementary Information.

## Data Availability

Data are not publicly available but may be requested from PICANet with appropriate permissions. Approvals were sought through the Healthcare Quality Improvement Partnership (HQI)P for the use of an anonymised extract of the PICANet data. All methods were carried out in accordance with the International Conference on Harmonisation Good Clinical Practice guidelines and in line with the Data Sharing Agreement with HQIP.
