# Supplementary material for: A study of sex difference in infant mortality in UK pediatric intensive care admissions over an 11-year period
Source: Sci Rep. 2021 Nov 8;11:21838. doi: 10.1038/s41598-021-01173-x (PMC8575897; doi:10.1038/s41598-021-01173-x)
Supplement: Supplementary file 1 — Supplementary Information. [file 41598_2021_1173_MOESM1_ESM.docx]

**Sex difference in infant mortality: a study of UK Pediatric Intensive Care admissions over 11 years**

**Supplementary Materials**

SUPPLEMENTARY PART A: **Causal diagrams**

The aim of the causal diagrams are to clarify the relationship between sex and mortality in PICU, in the presence of other causally linked variables. This is to ensure the correct adjustments are made in the analysis to avoid inducing a false association between sex and death in PICU.

The information incorporated in the causal graph may be incomplete and therefore the DAGs should be interpreted with caution. In particular our DAGs are based on a number of assumptions:

1. There is a different pattern between measured (age at admission, SES (IMD), ethnicity) and unmeasured factors (maternal and perinatal factors such as maternal illness during pregnancy, or complications during child birth or admission/contact with other health facilities) potentially affecting admission to PICU in that here measured factors are not directly caused by sex (only unmeasured factors may be).
2. There is no direct arrow between either measured and unmeasured preadmission factors and death in PICU as these factors are assumed to be directly linked to admission to PICU and be related to death only by influencing admission.
3. We have not included any time-varying (within PICU) variables such as information on duration of inotropic and renal support as we did not have information on their duration.

The three diagrams below show:

1. the unadjusted relationships between sex, mortality and all other variables
2. the relationship between the variables after conditioning on PICU admission, since there is a selection process leading to more males being admitted to PICU than females;
3. the relationship between variables after the additional adjustment for PIM2R.

A: unadjusted relationships between sex, mortality and all other variables


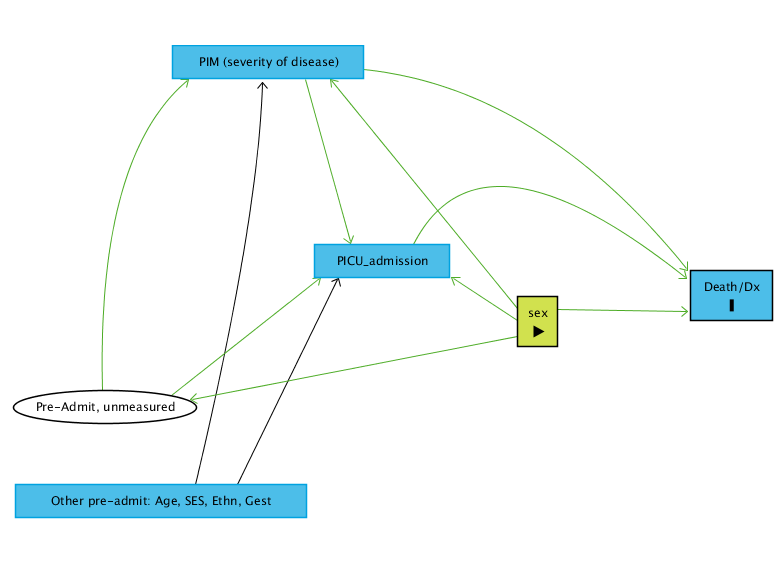

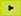
 Exposure


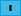
 Outcome


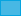
 Ancestor of outcome


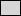
 Adjusted variable


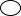
 Unobserved variable


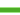
 Causal path


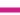
 Biasing path

Blocked path (can also be biasing paths after adjustment as seen in diagram C)

B: Relationship between the variables after conditioning on PICU admission


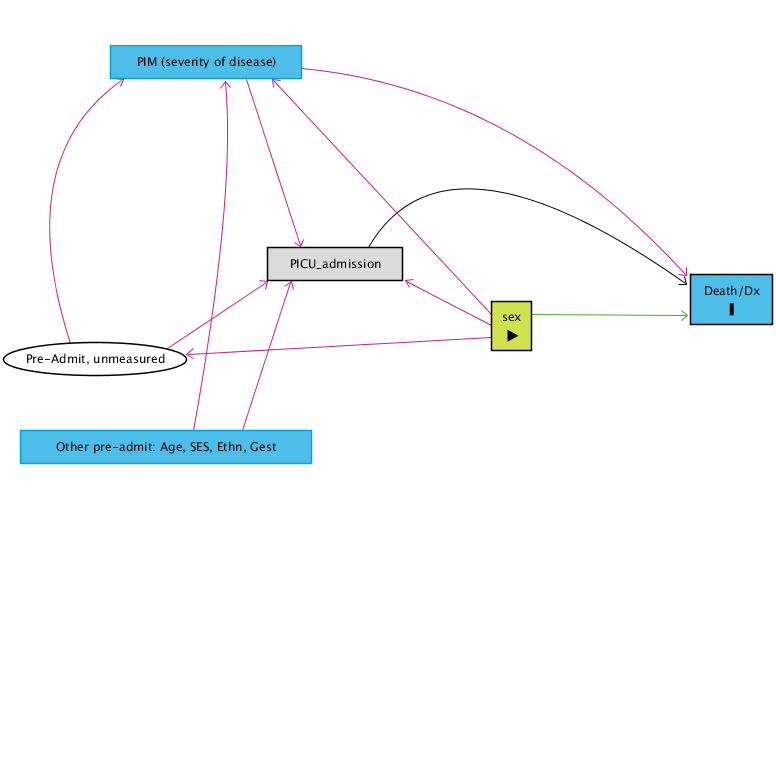


C: Relationship between variables after the additional adjustment for PIM2R


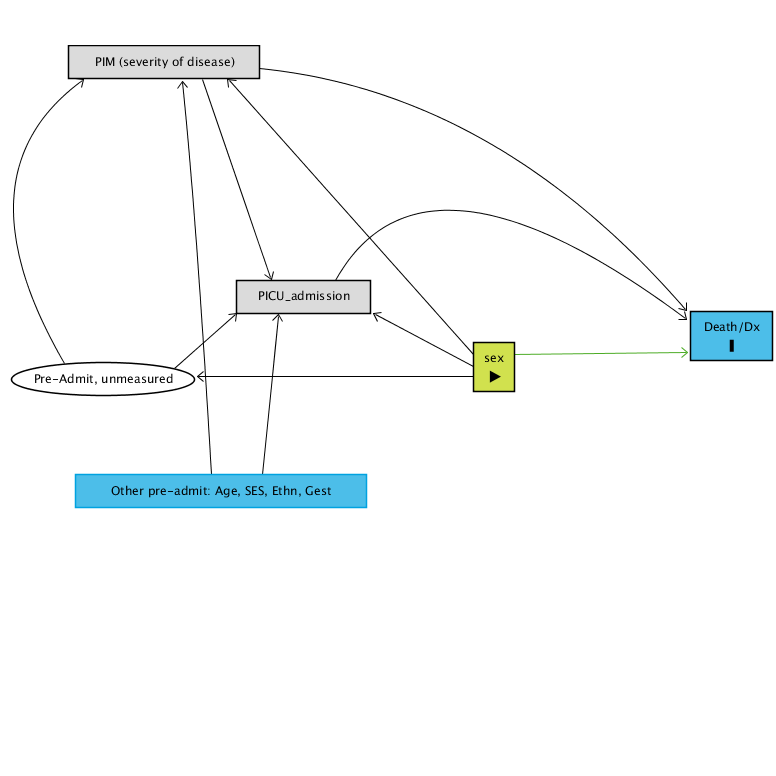


Diagram C shows that adjusting for PIM and not adjusting for age, ethnicity, SES, and gestation would not bias the relationship between sex and mortality in PICU, given the stated assumptions, thus allowing us to derive a valid estimate of the direct effect of sex on mortality in PICU.

In Diagrams A-C we assumed that there was no direct influence of the (measured or unmeasured) pre-admission factors on death in PICU. They assume that Death in PICU and discharge both occur only after admission to PICU and that the effect of all these factors on the outcomes are indirect as they only act by influencing the probability that a patient is admitted to PICU. However we also considered the causal estimation with a direct causal link between pre-admission factors (both measured and unmeasured) and death in PICU (DAGs D-G). We see in DAG D-G that if our main assumption on which DAG A-C are not correct, we would still be left with residual associations (red arrows) and therefore the adjustment would not be complete nor lead to a causally valid estimate.

D: DAG without adjustments


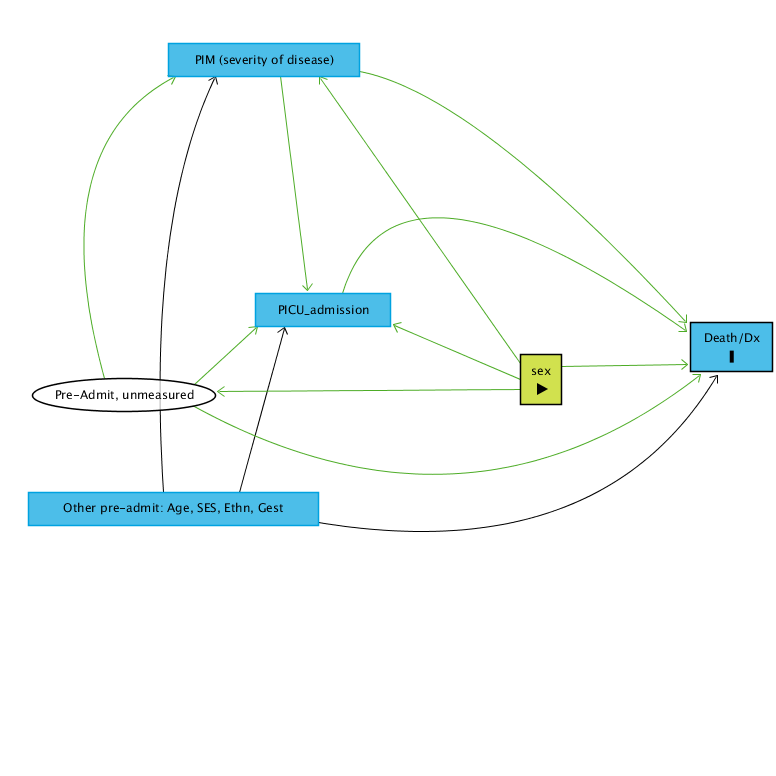


E: DAG Adjusted for admission, red lines are associations induced by the adjustment


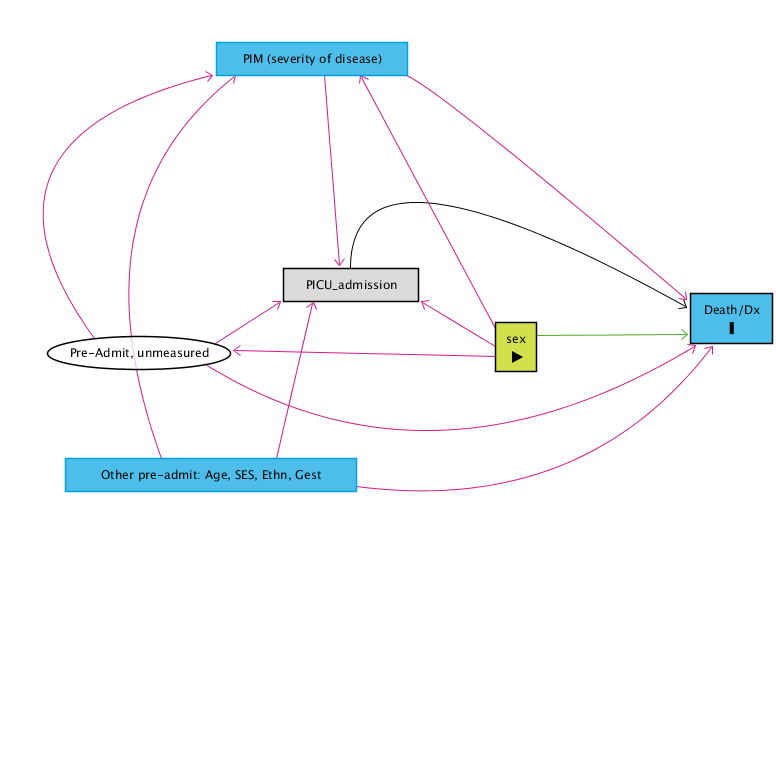


F: DAG Adjusted for PIM, some pathways are now blocked (black lines)


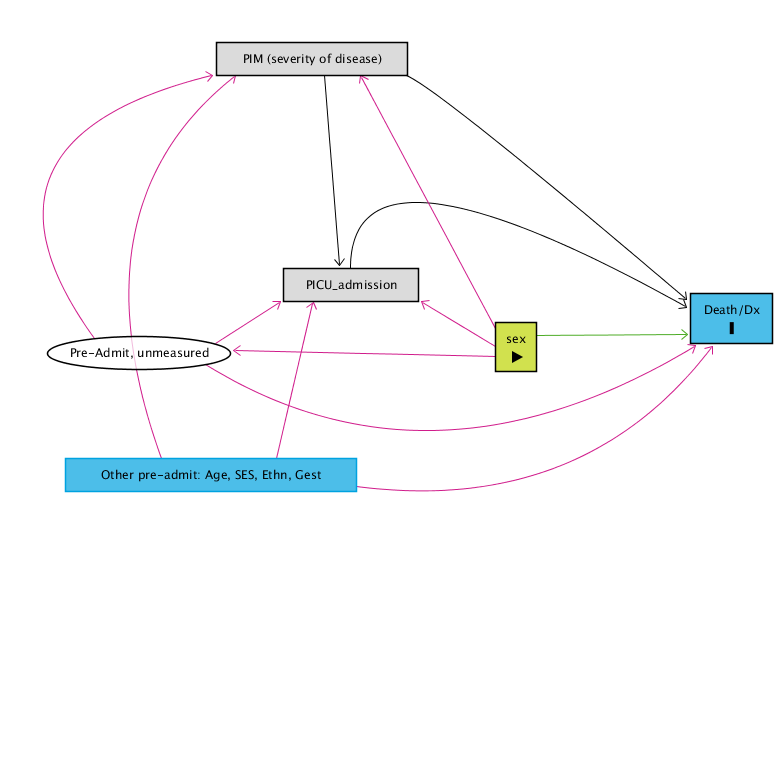


G: Further adjustment for observed pre-admission factors, some more pathways are blocked but residual associations remain.


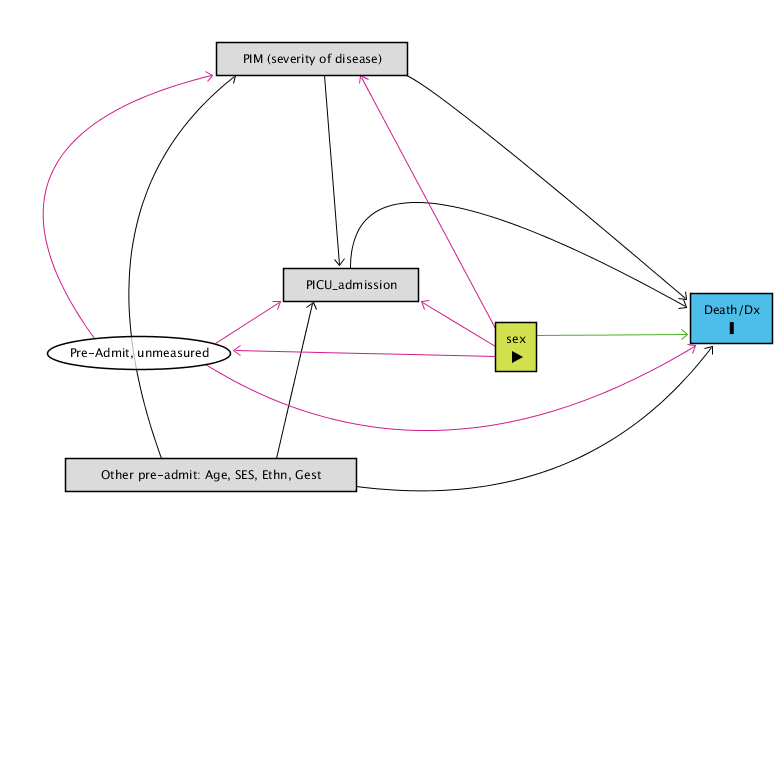


Code for causal diagram used in daggity^22^

This code may be copied and pasted into the daggity software to replicate the causal analysis that we have carried out. The daggity software is available from <http://www.dagitty.net/>.

dag {

"Death/Dx" [outcome,pos="1.024,0.363"]

"Other pre-admit: Age, SES, Ethn, Gest" [pos="0.175,0.600"]

"PIM (severity of disease)" [pos="0.406,0.122"]

"Pre-Admit, unmeasured" [latent,pos="0.107,0.456"]

PICU_admission [pos="0.455,0.367"]

sex [exposure,pos="0.765,0.391"]

"Other pre-admit: Age, SES, Ethn, Gest" -> "Death/Dx" [pos="0.675,0.669"]

"Other pre-admit: Age, SES, Ethn, Gest" -> "PIM (severity of disease)" [pos="0.341,0.266"]

"Other pre-admit: Age, SES, Ethn, Gest" -> PICU_admission

"PIM (severity of disease)" -> "Death/Dx" [pos="0.769,0.149"]

"PIM (severity of disease)" -> PICU_admission

"Pre-Admit, unmeasured" -> "Death/Dx" [pos="0.536,0.674"]

"Pre-Admit, unmeasured" -> "PIM (severity of disease)" [pos="0.177,0.223"]

"Pre-Admit, unmeasured" -> PICU_admission

PICU_admission -> "Death/Dx" [pos="0.688,0.177"]

sex -> "Death/Dx"

sex -> "PIM (severity of disease)"

sex -> "Pre-Admit, unmeasured"

sex -> PICU_admission

}

SUPPLEMENTARY PART B: **Sensitivity analyses**

1. We refitted the logistic model with the extreme LOS data (ie >100 days), with no change to the odds ratio for sex.
2. Assuming that causal diagrams D, E, F and G are the correct version, a logistic model was fitted including the pre-admission factors (Age, Ethnicity, Gestation, and SES). This model had a large number of missing data from the variables Ethnicity, Gestation, and SES, leading a sample size of 51% compared to the original. We assessed if the complete case analysis is valid using a procedure based on the article by Hughes et al^1^ (reported below in SUPPL PART C). We concluded that the complete case analysis was unbiased and the finding of higher mortality for females relative to males was virtually unchanged in magnitude (although due to non-collapsibility the effect estimate under DAG C and G are not directly comparable).
3. The Schoenfeld plots for proportional hazard assessment showed a potential curvature (ie non-proportional hazard) in the first few days of PICU admission. We explored this further by modelling sex with restricted cubic splines.

^1^Hughes, R. A., Heron, J., Sterne, J. A. C., *et al*. Accounting for missing data in statistical analyses: multiple imputation is not always the answer. *Int. J. Epidemiol.* **48**, 1294–1304 (2019).

SUPPLEMENTARY PART C: **Investigation of missingness and complete case analysis under DAG G**

The variables IMD score (SES), gestational age and ethnicity have missing data. Including these three variables for adjustment in our models reduces the model sample size to 36,303 which is 51% of the original size. The first issue that we would have with this is that our estimate for the effect of sex on mortality would be less precise. The second and more serious issue is that the missing data could lead to a biased estimate of the effect of sex on mortality. To assess this we followed the methods described by Hughes et al, 2019^1^.

For each incomplete variable in turn;

1. We fit a missingness model for being a complete case including said incomplete variable and all completely observed variables (sex, PIM2R) as predictors.
2. We generate a missingness variable “*_miss” from the estimation sample, for example IMD_miss, gestation_miss.
3. We fit a missingness model where the predictors are the complete variable PIM2R and death, and the variable *_miss identified from the first stage as predictive of being a complete case.
4. In all logistic models we accounted for clustering by PICU

The assessment showed that the CCA was unbiased, although less precise (TABLE S0), as expected. There were no dependencies of the probability of being missing of any of the three variables (IMD score, ethnicity, and gestation) on either the outcome (death) or the exposure (sex).

The odds ratio for sex in the CCA was 0·92, 95% CI 0·83 to 1·01. The direction of the effect is consistent with the main analyses and conclusions of higher female over male mortality.

SUPPLEMENTARY PART D: **Posterior probability calculations**

Since, conditionally on admission to PICU, we have:

Pr(Death in PICU|Female) = 0.048 (crude female mortality in PICU)

Pr(Death in PICU|Male) = 0.043 (crude male mortality in PICU)

And

Pr(Female) = 0.414

Pr(Male) = 0.586

Then by Bayes theorem one can work out the posterior sex probability ratio (odds) for those who die in PICU by deriving the respective conditional posterior probabilities:

Pr(Female|Death in PICU)=Pr(Death in PICU|Female)Pr(Female)/Pr(Death in PICU) = 0.048 x 0.414/(0.048 x 0.414 + 0.043 x 0.586) = 0.441

Pr(Male|Death in PICU)= Pr(Death in PICU|Male)Pr(Male)/Pr(Death in PICU) = 0.043 x 0.586/(0.048 x 0.414 + 0.043 x 0.586) = 0.559

Hence the posterior odds of being female vs male among PICU deaths is 0.441/0.559=0.79 and hence in favour of females, consistently with the published mortality rate ratio in the general population of children of that age (= 0.82, according to National Statistics figures)

**SUPPLEMENTARY TABLES**

| **Table S0. Parameters from the logistic model with complete case analysis (n = 36,303)** | | | | | |
| --- | --- | --- | --- | --- | --- |
|  | OR | SE | P | 95% CI | |
| Sex (male/female) | 0·918 | 0·045 | 0·079 | 0·835 | 1·010 |
| PIM2R | 1·079 | 0·003 | <0·001 | 1·072 | 1·086 |
| IMD2010 score | 1·003 | 0·002 | 0·026 | 1·000 | 1·007 |
|  |  |  |  |  |  |
| Ethnicity | (baseline white) | | | |  |
| Black | 0·999 | 0·118 | 0·993 | 0·793 | 1·258 |
| Asian | 1·644 | 0·152 | <0·001 | 1·371 | 1·970 |
| Mixed/Other | 1·142 | 0·110 | 0·168 | 0·946 | 1·378 |
|  |  |  |  |  |  |
| Gestational age | (baseline 36 weeks) | | |  |  |
| Up to 28 weeks | 1·203 | 0·222 | 0·315 | 0·838 | 1·727 |
| 29 to 35 weeks | 0·993 | 0·127 | 0·955 | 0·773 | 1·275 |
|  |  |  |  |  |  |
| Constant | 0·021 | 0·005 | <0·001 | 0·014 | 0·032 |

The Cause Specific Hazard Ratio model

| **Table S1. Parameters from the Cause-Specific Hazard Ratio model** | | | | | |
| --- | --- | --- | --- | --- | --- |
|  | CSHR | SE | P | 95% CI | |
| Sex (male/female) | 0·865 | 0·028 | <0·001 | 0·812 | 0·922 |
| PIM2R | 1·044 | 0·001 | <0·001 | 1·042 | 1·047 |
|  |  |  |  |  |  |
| Spline terms |  |  |  |  |  |
| Baseline 1 | -0·082 | 0·022 | <0·001 | -0·124 | -0·039 |
| Baseline 2 | -0·135 | 0·020 | <0·001 | -0·175 | -0·095 |
| Baseline 3 | -0·049 | 0·012 | <0·001 | -0·073 | -0·026 |
| Baseline 4 | 0·132 | 0·016 | <0·001 | 0·100 | 0·165 |
| PIM2R 1 | -0·008 | 0·001 | <0·001 | -0·010 | -0·007 |
| PIM2R 2 | 0·003 | 0·001 | <0·001 | 0·002 | 0·004 |
| PIM2R 3 | -0·001 | 0·000 | 0·034 | -0·002 | 0·000 |
| PIM2R 4 | -0·002 | 0·001 | <0·001 | -0·003 | -0·001 |
| Constant | -5·038 | 0·076 | <0·001 | -5·187 | -4·888 |
| CI: Confidence interval; P: p value; SE: Standard error | | | | | |

Subgroup analyses

| **Table S2. OR Subgroup analysis of children admitted under 56 days of age (n=33611)** | | | | | |  |
| --- | --- | --- | --- | --- | --- | --- |
|  | OR | SE | P | 95% CI | | |
| Sex (male/female) | 0·839 | 0·048 | 0·002 | 0·749 | 0·939 | |
| PIM2R | 1·075 | 0·004 | 0·000 | 1·068 | 1·082 | |
| Constant | 0·049 | 0·005 | 0·000 | 0·041 | 0·060 | |

CI: Confidence interval; OR: Odds ratio; P: p value; SE: Standard error

| **Table S3. OR Subgroup analysis of children admitted with primary diagnosis of infection (n=8402)** | | | | | |
| --- | --- | --- | --- | --- | --- |
|  | OR | SE | P | 95% CI | |
| Sex (male/female) | 0·874 | 0·082 | 0·152 | 0·728 | 1·051 |
| PIM2R | 1·068 | 0·008 | 0·000 | 1·053 | 1·084 |
| Constant | 0·051 | 0·006 | 0·000 | 0·041 | 0·063 |

CI: Confidence interval; OR: Odds ratio; P: p value; SE: Standard error

| **Table S4. OR Subgroup analysis of children older than 56 days (n=37435)** | | | | | |
| --- | --- | --- | --- | --- | --- |
|  | OR | SE | P | 95% CI | |
| Sex (male/female) | 0·877 | 0·043 | 0·008 | 0·796 | 0·966 |
| PIM2R | 1·095 | 0·005 | 0·000 | 1·085 | 1·106 |
| Constant | 0·030 | 0·002 | 0·000 | 0·025 | 0·035 |

CI: Confidence interval; OR: Odds ratio; P: p value; SE: Standard error

| **Table S5. OR Subgroup analysis of children admitted with primary diagnosis of no infection (n=62644)** | | | | | |
| --- | --- | --- | --- | --- | --- |
|  | OR | SE | P | 95% CI | |
| Sex (male/female) | 0·856 | 0·042 | 0·002 | 0·777 | 0·944 |
| PIM2R | 1·087 | 0·003 | 0·000 | 1·080 | 1·093 |
| Constant | 0·037 | 0·003 | 0·000 | 0·031 | 0·044 |

CI: Confidence interval; OR: Odds ratio; P: p value; SE: Standard error

| **Table S6. CSHR Subgroup analysis of children younger than 56 days (n=33687)** | | | | | |
| --- | --- | --- | --- | --- | --- |
|  | CSHR | SE | P | 95% CI | |
| Sex (male/female) | 0·848 | 0·042 | 0·001 | 0·769 | 0·934 |
| PIM2R | 1·041 | 0·001 | 0·000 | 1·038 | 1·044 |
|  |  |  |  |  |  |
| Spline terms |  |  |  |  |  |
| Baseline 1 | -0·084 | 0·030 | 0·006 | -0·143 | -0·025 |
| Baseline 2 | -0·128 | 0·022 | <0·001 | -0·171 | -0·085 |
| Baseline 3 | -0·037 | 0·017 | 0·031 | -0·072 | -0·003 |
| Baseline 4 | 0·152 | 0·020 | <0·001 | 0·113 | 0·191 |
| PIM2R 1 | -0·009 | 0·001 | <0·001 | -0·011 | -0·007 |
| PIM2R 2 | 0·002 | 0·001 | 0·001 | 0·001 | 0·004 |
| PIM2R 3 | -0·001 | 0·001 | 0·049 | -0·003 | 0·000 |
| PIM2R 4 | -0·002 | 0·001 | 0·091 | -0·003 | 0·000 |
| Constant | -4·830 | 0·081 | <0·001 | -4·989 | -4·672 |

CI: Confidence interval; CSHR: Cause specific hazard ratio; P: p value; SE: Standard error

| **Table S7. CSHR Subgroup analysis of children older than 56 days (n=37547)** | | | | | |
| --- | --- | --- | --- | --- | --- |
|  | CSHR | SE | P | 95% CI | |
| Sex (male/female) | 0·876 | 0·047 | 0·014 | 0·789 | 0·974 |
| PIM2R | 1·048 | 0·001 | <0·001 | 1·046 | 1·050 |
|  |  |  |  |  |  |
| Spline terms |  |  |  |  |  |
| Baseline 1 | -0·070 | 0·030 | 0·019 | -0·128 | -0·011 |
| Baseline 2 | -0·158 | 0·027 | <0·001 | -0·211 | -0·104 |
| Baseline 3 | -0·064 | 0·015 | <0·001 | -0·094 | -0·035 |
| Baseline 4 | 0·116 | 0·022 | <0·001 | 0·074 | 0·159 |
| PIM2R 1 | -0·008 | 0·001 | <0·001 | -0·010 | -0·006 |
| PIM2R 2 | 0·004 | 0·001 | <0·001 | 0·003 | 0·005 |
| PIM2R 3 | -0·001 | 0·001 | 0·222 | -0·002 | 0·000 |
| PIM2R 4 | -0·003 | 0·001 | <0·001 | -0·004 | -0·001 |
| Constant | -5·310 | 0·076 | <0·001 | -5·459 | -5·162 |

CI: Confidence interval; CSHR: Cause specific hazard ratio; P: p value; SE: Standard error

| **Table S8. CSHR Subgroup analysis of children admitted with primary diagnosis of infection (n=8411)** | | | | | |
| --- | --- | --- | --- | --- | --- |
|  | CSHR | SE | P | 95% CI | |
| Sex (male/female) | 0·887 | 0·076 | 0·163 | 0·751 | 1·049 |
| PIM2R | 1·039 | 0·004 | <0·001 | 1·031 | 1·047 |
|  |  |  |  |  |  |
| Spline terms |  |  |  |  |  |
| Baseline 1 | -0·203 | 0·040 | <0·001 | -0·281 | -0·124 |
| Baseline 2 | -0·120 | 0·049 | 0·016 | -0·216 | -0·023 |
| Baseline 3 | -0·128 | 0·026 | <0·001 | -0·179 | -0·078 |
| Baseline 4 | 0·058 | 0·037 | 0·111 | -0·013 | 0·130 |
| PIM2R 1 | -0·011 | 0·002 | <0·001 | -0·015 | -0·006 |
| PIM2R 2 | 0·006 | 0·002 | 0·003 | 0·002 | 0·010 |
| PIM2R 3 | 0·004 | 0·002 | 0·033 | 0·000 | 0·007 |
| PIM2R 4 | 0·003 | 0·002 | 0·051 | 0·000 | 0·007 |
| Constant | -4·768 | 0·084 | <0·001 | -4·933 | -4·602 |

CI: Confidence interval; CSHR: Cause specific hazard ratio; P: p value; SE: Standard error

| **Table S9. CSHR Subgroup analysis of children admitted with primary diagnosis of no infection (n=62823)** | | | | | |
| --- | --- | --- | --- | --- | --- |
|  | CSHR | SE | P | 95% CI | |
| Sex (male/female) | 0·862 | 0·036 | <0·001 | 0·794 | 0·936 |
| PIM2R | 1·045 | 0·001 | <0·001 | 1·043 | 1·047 |
|  |  |  |  |  |  |
| Spline terms |  |  |  |  |  |
| Baseline 1 | -0·055 | 0·024 | 0·021 | -0·101 | -0·008 |
| Baseline 2 | -0·140 | 0·023 | <0·001 | -0·186 | -0·094 |
| Baseline 3 | -0·037 | 0·013 | 0·005 | -0·063 | -0·011 |
| Baseline 4 | 0·141 | 0·017 | <0·001 | 0·107 | 0·176 |
| PIM2R 1 | -0·008 | 0·001 | <0·001 | -0·010 | -0·007 |
| PIM2R 2 | 0·003 | 0·001 | <0·001 | 0·002 | 0·004 |
| PIM2R 3 | -0·001 | 0·001 | 0·021 | -0·002 | 0·000 |
| PIM2R 4 | -0·002 | 0·001 | <0·001 | -0·003 | -0·001 |
| Constant | -5·093 | 0·084 | <0·001 | -5·257 | -4·929 |

CI: Confidence interval; CSHR: Cause specific hazard ratio; P: p value; SE: Standard error
